# Real‐world effectiveness and safety of RC48‐ADC alone or in combination with PD‐1 inhibitors for patients with locally advanced or metastatic urothelial carcinoma: A multicenter, retrospective clinical study

**DOI:** 10.1002/cam4.6680

**Published:** 2023-11-07

**Authors:** Jingwei Xu, Hongqiao Zhang, Li Zhang, Xiufeng Chu, Yu Li, Guangyuan Li, Caiyun Nie, Meng Wang, Yanwei Guo

**Affiliations:** ^1^ Department of Oncology The Fifth Affiliated Hospital of Zhengzhou University Zhengzhou China; ^2^ Department of Oncology Henan Cancer Hospital, Affiliated Cancer Hospital of Zhengzhou University Zhengzhou China; ^3^ Department of Radiology The First Affiliated Hospital of Zhengzhou University Zhengzhou China; ^4^ Department of Oncology The Second Affiliated Hospital of The Chinese University of Hong Kong, Shenzhen & Longgang District People's Hospital of Shenzhen Shenzhen China

**Keywords:** antibody–drug conjugate, disitamab vedotin, metastatic urothelial carcinoma, real‐world study

## Abstract

**Introduction:**

Previous RC48 (Disitamab Vedotin) studies established that the safety and efficacy of RC48‐antibody–drug conjugate (ADC), either alone or combined with toripalimab, for metastatic urothelial carcinoma (mUC) patients exhibiting human epidermal growth factor receptor 2 (HER2)‐positive or even HER2‐negative status after standard chemotherapy failure.

**Methods:**

With locally advanced or metastatic urothelial carcinoma (la/mUC), patients who received RC48‐ADC monotherapy or a combination with programmed cell death protein 1 (PD‐1) inhibitors between August 2021 and October 2022 were enrolled in this retrospective observational study to evaluate the real‐world antitumor effectiveness and safety.

**Results:**

Among the 38 enrolled patients (29 males; median age 67.5 years [38–93]), 8 received RC48‐ADC monotherapy, while 30 received combination therapy. Initially, 63.2% (24/38) of the patients had received ≥1 line of prior treatment, and 63.2% (24/38) had visceral metastasis. UC of the bladder represented the majority type in 68.4% (26/38) of cases. By the data cutoff in March 2023, the overall objective response rate (ORR) was 63.2% (95% CI, 47.1%–79.2%), with a disease control rate (DCR) of 89.5% (95% CI, 79.3%–99.7%). Median follow‐up time was 10.6 months. The median progression‐free survival (PFS) was 8.2 months (95% CI, 5.9–10.5), with a 6‐month PFS rate of 63.2% and a 12‐month PFS rate of 34.1%. Median overall survival (OS) was not reached, with a 12‐month OS rate of 76.7%. The median duration of response was 7.3 months (95% CI, 4.6–10.0) among 24 patients evaluated as partial response (PR). The most common treatment‐related adverse events (TRAEs) included anemia (71.1%), anorexia (57.9%), asthenia (52.6%), hypoesthesia (52.6%), bone marrow suppression (47.4%), alopecia (47.4%), nausea (44.7%), proteinuria (36.8%), vomiting (34.2%), and hypoalbuminemia (31.6%). No patient experienced TRAEs of Grade ≥3. One patient had an immune‐related adverse event (irAE) of rash related to toripalimab.

**Conclusions:**

Both as monotherapy and in combination with PD‐1 inhibitors, RC48‐ADC exhibits promising effectiveness and manageable safety profile for mUC patients in real‐world settings.

## 1 INTRODUCTION

Urothelial carcinoma (UC) prognosis worsens with tumor progression. The 5‐year survival rate plunges to 20.9% for patients with only lymph node metastasis and a mere 6.8% for those with visceral metastasis.^1^ In 2022, statistics projected approximately 92,000 new bladder cancer cases in China, leading to about 43,000 deaths. Influenced by the aging population and urbanization, both incidence and mortality rates of bladder cancer in China are projected to rise further.^2^, ^3^ Currently, platinum‐based combination chemotherapy is the standard treatment for locally advanced or metastatic UC (la/mUC), offering a median progression‐free survival (mPFS) of 7.6–8.3 months.^1^, ^4^ Following the results from the Polaris‐03 and BGB‐A317‐204 studies, immune checkpoint inhibitors (ICIs) toripalimab and tislelizumab were approved as second‐line treatments for mUC, establishing a new standard for second‐line treatment in China. However, only a limited proportion of patients respond to ICIs, as toripalimab and tislelizumab exhibiting objective response rates (ORRs) of 25.2% and 24.0%, respectively.^5^, ^6^

In recent years, advancements in genomic research have identified numerous promising targets for UC, marking the advent of the targeted therapy era for this disease. These targeted therapy agents include erdafitinib (targeting fibroblast growth factor receptor, FGFR), vofatamab (targeting FGFR), and various antibody–drug conjugates (ADCs), such as enfortumab vedotin targeting Nectin‐4, sacituzumab govitecan targeting Trop‐2, and RC48‐ADC (Disitamab Vedotin, DV) targeting human epidermal growth factor receptor 2 (HER2).^7^, ^8^, ^9^, ^10^, ^11^, ^12^, ^13^, ^14^

RC48‐ADC is a novel humanized anti‐HER2 ADC.^15^ Recently conducted Phase II multicenter randomized controlled studies, namely RC48‐C005, RC48‐C009, and RC48‐C011, demonstrated robust ORR and PFS data for UC treated with RC48‐ADC monotherapy, regardless of HER2 expression (IHC 3+, 2+, 1+, or 0), after progression on standard chemotherapy.^16^, ^17^, ^18^ The latest RC48‐C014 study confirmed that the combination of RC48‐ADC with the programmed cell death protein 1 (PD‐1) inhibitor toripalimab outperformed RC48‐ADC monotherapy in terms of ORR and median PFS.^19^

A combined analysis of the RC48‐C005 and RC48‐C009 studies revealed that RC48‐ADC monotherapy achieved an ORR of 50.5% (95% CI: 40.6%–60.3%) in HER2‐positive (IHC2+ and 3+) mUC patients who had progressed on at least one systemic chemotherapy. The disease control rate (DCR) was 82.2% (95% CI: 73.7%, 89.0%), mPFS of 5.9 months (95% CI: 4.2, 7.2) and a median overall survival (mOS) of 14.2 months (95% CI: 9.7, 18.8).^17^ The RC48‐C011 study demonstrated that RC48‐ADC monotherapy achieved an ORR of 26.3% (95% CI: 9.1%, 51.2%) in la/mUC patients with low HER2 expression (IHC1+ and 0), a DCR of 94.7%, an mPFS of 5.5 months (95% CI: 3.9, 6.8), and an mOS of 16.4 months (95% CI: 7.1, 21.7).^16^ The RC48‐C014 study showed an ORR of 76.7% (95% CI: 57.7%–90.1%), an mPFS of 9.2 months, and an unreached mOS for RC48‐ADC combined with toripalimab in la/mUC patients.^19^ This retrospective study sought to evaluate the real‐world efficacy and safety of RC48‐ADC, either alone or in combination with PD‐1 inhibitors, for treating patients with la/mUC. The study aims to provide valuable insights to inform treatment strategies for these patients.

## 2 MATERIALS AND METHODS

### 2.1 Study design and patients

This was a retrospective, multicenter, real‐world study that collected data via a web‐based electronic medical record system from patients with la/mUC who underwent treatment with RC48 monotherapy or in combination with PD‐1 inhibitors at the Fifth Affiliated Hospital of Zhengzhou University (Zhengzhou, China), the First Affiliated Hospital of Zhengzhou University (Zhengzhou, China), and the Henan Cancer Hospital (Zhengzhou, China) between August 2021 and October 2022. Recorded data included age, gender, histological differentiation, primary lesion site, Eastern Cooperative Oncology Group Performance Status (ECOG PS), smoking history, previous immunotherapy, metastatic lesion site, presence of visceral metastasis, surgery status, HER2 expression status, number of lines of RC48 treatment, and treatment regimen. All data were stored in an irreversibly anonymized form.

No limitations of lines of prior therapy, and all levels of PD‐L1 and HER2 expression are allowed. The inclusion criteria were as follows:

(1) patients with la/mUC presenting at least one measurable lesion;

(2) patients who received either RC48 monotherapy or RC48‐ADC combined with PD‐1 inhibitors, and had undergone treatment effectiveness evaluation.

This study's procedures complied with the ethical standards outlined in the World Medical Association Declaration of Helsinki.^20^ All patients provided informed consent before treatment.

RC48 dose cited in this article is based on calculations using bovine serum albumin (BSA)‐based extinction coefficient (EC) implemented in China. Outside of China, RC48 dose calculation is based on DV EC which is equivalent to 1.07 (BSA‐based EC)/1.41 (DV‐based EC) × BSA‐based EC dose.

### 2.2 Follow‐up process

Follow‐up was performed by medical records review, outpatient follow‐ups, and telephone contacts starting from the application of RC48 monotherapy or combination with PD‐1 inhibitors, unless the patient died, progressed due to the disease, could not tolerate treatment‐related adverse events (TRAEs), or voluntarily discontinued treatment. And the follow‐up period was up to March 31, 2023. Any alterations in dosage during treatment were recorded for patients who were on the standard dose of RC48 (2.0 mg/kg), tislelizumab (200 mg), and toripalimab (3 mg/kg). The administration of PD‐1 inhibitors was decided by the treating clinicians. The expression status of HER2 was primarily determined through immunohistochemistry (IHC) tests, with the IHC score assessed following the HER2 breast cancer testing guidelines.^21^

### 2.3 Endpoints and response definitions

The researchers assessed the antitumor therapeutic efficacy in accordance with the Response Evaluation Criteria in Solid Tumors (RECIST) version 1.1. Adverse events were monitored and categorized based on the Common Terminology Criteria for Adverse Events (CTCAE) 5.0.

An objective response was specified as a reaction persisting for a minimum of two consecutive imaging assessments with an interval of at least 4 weeks. Various imaging evaluations such as CT scans of primary and metastatic lesions, bone scans, and magnetic resonance imaging (MRI) were routinely conducted before and during treatment every 6 weeks. Before each treatment cycle, liver and kidney functions, routine blood tests, biochemistry, coagulation functions, tumor markers, and electrocardiograms were rechecked. The exact tests conducted were decided by the clinical physician based on the patient's individual situation.

From the collected medical record data, observed indicators included ORR, PFS, DCR, overall survival (OS), duration of response (DOR), 6‐month PFS rate, 12‐month PFS rate, 12‐month survival rate, safety, and dose adjustment. The ORR was calculated as the combined total of complete response (CR) and partial response (PR) rates. The DCR was calculated as the percentage of cases showing CR, PR, and stable disease (SD) among the evaluable cases posttreatment.

### 2.4 Statistical analysis

Statistical analysis was conducted using SPSS 27.0 software and GraphPad Prism 9.0. Descriptive statistics, such as percentages, means, and medians, were used to report the patients' baseline characteristics and adverse events. Fisher's exact test was applied for the analysis of categorical variables. The Kaplan–Meier method was used to perform survival analysis, and the log‐rank test was used for comparison of survival between groups. Multivariate analysis of patient progression‐free survival (PFS) was conducted using the Cox proportional hazards regression model. Differences were considered statistically significant when *p* < 0.05.

## 3 RESULTS

During the study period, six patients who met the inclusion criteria were excluded due to a loss of follow‐up. The study utilized data from 38 patients with la/mUC who received RC48 monotherapy or in conjunction with PD‐1 inhibitors at the Fifth Affiliated Hospital of Zhengzhou University (*n* = 15), Henan Cancer Hospital (*n* = 11), and the First Affiliated Hospital of Zhengzhou University (*n* = 12). The demographic and baseline characteristics of these patients are detailed in Table 1.

**TABLE 1:** Baseline demographics and clinical features of 38 patients.

	RC48 + PD‐1 inhibitor (*n* = 30)	RC48 alone (*n* = 8)
Age (years)		
Median	70	65
Mean (SD)	67 (13.0)	63 (10.5)
Min, max	38, 93	49, 77
Gender, *n* (%)		
Male	23 (76.7%)	6 (75.0%)
Female	7 (23.3%)	2 (25.0%)
Primary site, *n* (%)		
Bladder	19 (63.3%)	7 (87.5%)
Renal pelvis	9 (30.0%)	1 (12.5%)
Ureter	5 (16.7%)	1 (12.5%)
Organizational differentiation, *n* (%)		
UC without differentiation	28 (93.3%)	8 (100.0%)
UC with squamous differentiation	2 (6.7%)	0
ECOG score, *n* (%)		
0	17 (56.7%)	2 (25.0%)
1	9 (30.0%)	3 (37.5%)
2	4 (13.3%)	3 (37.5%)
Smoking history, *n* (%)		
No	25 (83.3%)	5 (62.5%)
Yes	5 (16.7%)	3 (37.5%)
Underlying disease, *n* (%)		
No	11 (36.7%)	2 (25.0%)
Yes	19 (63.3%)	6 (75.0%)
Hypertension	13 (43.3%)	3 (37.5%)
Coronary heart disease	9 (30.0%)	1 (12.5%)
Diabetes	5 (16.7%)	2 (25.0%)
Hypothyroidism	3 (10.0%)	0
Pancreatitis	0	1 (12.5%)
Atrial fibrillation	1 (3.3%)	0
Previous immunotherapy, *n* (%)		
No	16 (53.3%)	4 (50.0%)
Yes	14 (46.7%)	4 (50.0%)
Tislelizumab	9 (30.0%)	4 (50.0%)
Toripalimab	4 (13.3%)	0
Camrelizumab	3 (10.0%)	0
HER2 (IHC), *n* (%)		
Uncertain^a^	18 (60.0%)	2 (25.0%)
2+/3+	9 (30.0%)	5 (62.5%)
0/1+	3 (10.0%)	1 (12.5%)
Metastasis sites, *n* (%)		
Lymph nodes	18 (60.0%)	5 (62.5%)
Lung	12 (40.0%)	3 (37.5%)
Bone	10 (33.3%)	3 (37.5%)
Liver	7 (23.3%)	1 (12.5%)
Pelvic cavity	6 (20.0%)	3 (37.5%)
Muscles	6 (20.0%)	1 (12.5%)
Spleen	3 (10.0%)	0
The primary lesion recurs	2 (6.7%)	1 (12.5%)
Adrenal gland	2 (6.7%)	0
Kidney	2 (6.7%)	0
Ureter	2 (6.7%)	0
Surgery or not, *n* (%)		
Yes	28 (93.3%)	8 (100.0%)
Radical resection	21 (70.0%)	6 (75.0%)
Local resection	7 (23.3%)	2 (25.0%)
No	2 (6.7%)	0
RC48 treatment lines, *n* (%)		
1	12 (40.0%)	2 (25.0%)
2	11 (36.7%)	6 (75.0%)
≥3	7 (23.3%)	0
Combined treatment regimen, *n* (%)		
Tislelizumab	18 (60.0%)	–
Toripalimab	12 (40.0%)	–

*Note*: Data are presented as *n* (%).

Abbreviations: ECOG PS, Eastern Cooperative Oncology Group Performance Status; HER2, human epidermal growth factor receptor 2; IHC, immunohistochemistry; SD, standard deviation; UC, urothelial carcinoma.

^a^ We could only find the patients' electronic medical records that “in combination with HER2 expression results and relevant clinical trial data, RC48‐ADC (Disitamab Vedotin) was recommended, and after detailed communication with the patients and their families, they agreed and signed the informed consent forms.”

Among the patients, 8 received RC48‐ADC monotherapy, while 30 received RC48‐ADC in combination with PD‐1 inhibitors. At the outset, 63.2% (24/38) had previously received at least one treatment line. Similarly, 63.2% (24/38) had visceral metastases, with 15 cases (39.5%) of lung metastasis, 8 cases (21.1%) of liver metastasis, 3 cases (7.9%) of spleen metastasis, 2 cases (5.3%) of adrenal gland metastasis, and 2 cases (5.3%) of kidney metastasis. The predominant primary site was bladder UC, making up 68.4% (26/38). Eighteen patients (47.4%) had received prior PD‐1 inhibitor treatment, with 13 cases involving tislelizumab, 4 cases of toripalimab, and 3 cases of camrelizumab. A majority of patients (94.7%) underwent surgery, with 27 (71.1%) radical resections and 9 (23.7%) local resections. However, two (5.3%) patients did not receive surgery.

**FIGURE 1:**
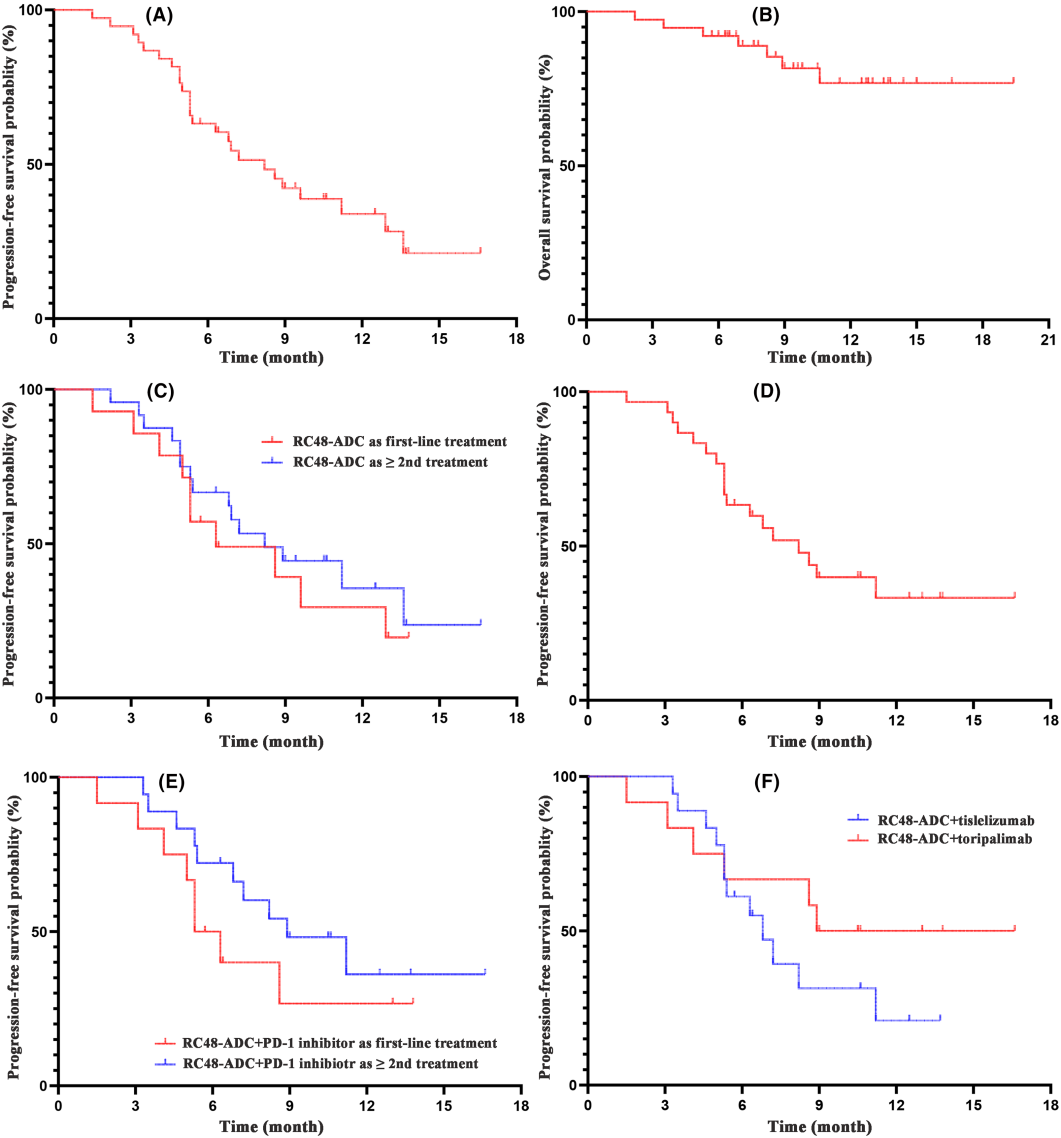
(A) PFS and (B) OS curves for all patients. (C) PFS curves for patients who received RC48 as the first‐line treatment group and ≥2nd‐line treatment group, respectively. (D) PFS curve for patients who received a combination therapy of RC48‐ADC and PD‐1 inhibitors. (E) PFS curves for patients who received combination therapy as the first‐line treatment group and ≥2nd‐line treatment group, respectively. (F) PFS curves for patients who received different combination therapies.

**FIGURE 2:**
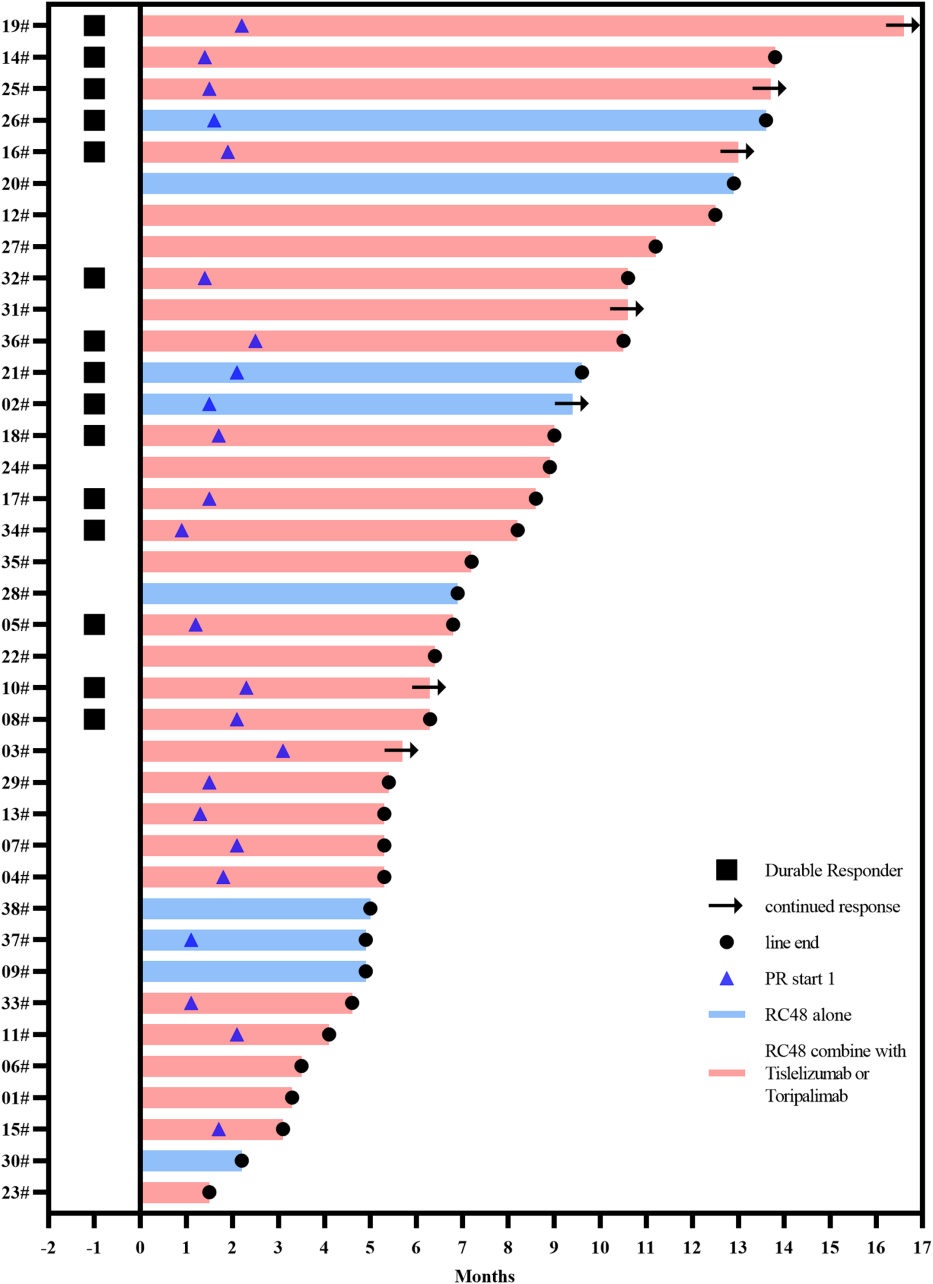
Swimmer plot for response evaluation. Start 1 means the time when the patient was first evaluated as PR.

As of March 31, 2023, the median follow‐up time for patients was 10.6 months (ranging between 2.2 and 19.4 months). All patients had at least one response evaluation (Table 2). According to RECIST 1.1, objective responses were confirmed in 24 patients, including 0 CR and 24 (63.2%) PR, and 10 (26.3%) patients had SD as the best response. The ORR was 63.2% (95% CI, 41.7%–79.2%; Table 2) and the DCR was 89.5% (95% CI, 79.3%–99.7%; Table 2). The mPFS was 8.2 months (95% CI, 5.9–10.5; Table 2; Figure 1A), with a 6‐month PFS rate of 63.2% and a 12‐month PFS rate of 34.1%. The mOS was not achieved (Figure 1B), and the 1‐year OS rate was 76.7%. The median DOR (mDOR) of 24 patients evaluated for PR was 7.3 months (95% CI, 4.6–10.0; Table 2; Figure 2).

**TABLE 2:** Tumor response evaluation of 38 patients.

	*n* = 38
BOR, *n* (%; 95% CI)	24 (63.2%;47.1%–79.2%)
Complete response	0
Partial response	24 (63.2%;47.1%–79.2%)
Stable disease	10 (26.3%;11.6%–41.0%)
Progressive disease	4 (10.5%;0.3%–20.7%)
Confirmed ORR (95% CI)	63.2% (47.1%–79.2%)
RC48 as first‐line treatment	71.4% (44.4%–98.5%)
RC48 as ≥2nd line treatment	58.3% (37.1%–79.6%)
RC48 + PD‐1 inhibitor	66.7% (48.8%–84.6%)
RC48 alone	50.0% (5.3%–94.7%)
HER2 IHC (2+ and 3+)	71.4% (44.4%–98.5%)
HER2 IHC 1+ and 0)	50.0% (5.3%–94.7%)
HER2 uncertain^a^	60.0% (36.5%–83.5%)
DCR	89.5% (79.3%–99.7%)
Duration of response, months (95% CI)	7.3 (4.6–10.0)

*Note*: Data are presented as *n* (%).

Abbreviations: BOR, best overall response; CI, confidence interval; DCR, disease control rate; HER2, human epidermal growth factor receptor 2; IHC, immunohistochemistry; ORR, objective response rate; PD‐1, programmed cell death protein 1; RC48, Disitamab Vedotin.

^a^ We could only find the patients' electronic medical records that “in combination with HER2 expression results and relevant clinical trial data, RC48‐ADC (Disitamab Vedotin) was recommended, and after detailed communication with the patients and their families, they agreed and signed the informed consent forms.”

ORRs divided by pathological baseline characteristics are shown in Figure S1. Subgroup analysis demonstrated an ORR of 71.4% (10/14) for patients without prior treatment, and an ORR of 58.3% (14/24) for patients with at least one line of prior therapy. For patients without prior PD‐1 inhibitor treatment, the ORR was 70.0% (14/20), while for those with prior PD‐1 inhibitor therapy, the ORR was 55.6% (10/18). The ORR for patients with HER2 overexpression (IHC2+ and 3+) was 71.4% (10/14), and for patients with low HER2 expression (0/1+), the ORR was 50.0% (2/4). For patients with uncertain HER2 expression, the ORR was 60.0% (12/20). For patients with lung metastases, the ORR was 66.7% (10/15), and for patients with liver metastases, the ORR was 62.5% (5/8). Among the eight patients who underwent PD‐1 companion diagnostic testing, one with positive PD‐1 expression achieved PR, while the ORR was 57.1% (4/7) for those with negative PD‐1 expression. The ORR for the eight patients who received RC48 monotherapy was 50.0% (4/8), with a DCR of 87.5% (7/8). For the 30 patients who received RC48 combined with PD‐1 inhibitors, the ORR was 66.7% (95% CI, 48.8%–84.6%), with a DCR of 90.0% (27/30). Representative images of metastasis responses are depicted in Figure S2. Subgroup analysis showed numerically longer median PFS in the combination therapy group (8.2 months; Figure 1D) compared to the RC48‐ADC monotherapy group (6.9 months; HR = 1.10, 95% CI 0.31–3.93, *p* = 0.50). The median PFS for patients who received RC48‐ADC as first‐line treatment was 6.3 months (Figure 1C), while for those who received it as second‐line or subsequent treatment, it was 8.2 months (Figure 1C) (*p* = 0.49). The mPFS for patients with HER2 overexpression was 6.8 months, while for those with low HER2 expression, it was 5.4 months (*p* = 0.74).

In the combination group (*n* = 30), the ORR for the 13 patients who received first‐line treatment of RC48 with PD‐1 inhibitors was 76.9% (10/13), whereas for the 17 patients who received this regimen as second‐line or later, the ORR was 58.8% (10/17), with mPFS of 5.3 and 8.9 months, respectively (HR = 0.14, 95% CI 0.01–1.64, *p* = 0.23; Figure 1E). The ORR for the 18 patients treated with RC48‐ADC in combination with tislelizumab was 61.1% (11/18), while for the 12 patients treated with RC48‐ADC in combination with toripalimab, it was 75.0% (9/12). The latter group showed a numerically longer mPFS of 8.9 months, compared to 6.8 months for the former (HR = 0.52, 95% CI 0.10–2.79, *p* = 0.29; Figure 1F).

Table 3 lists the TRAEs in 38 patients. Given their advanced age and poor baseline characteristics, the patients experienced many TRAEs, but the severity was not high. The TRAEs with an incidence rate exceeding 30% included anemia (71.1%, 27/38), anorexia (57.9%, 22/38), asthenia (52.6%, 20/38), hypoesthesia (52.6%, 20/38), myelosuppression (47.4%, 18/38), alopecia (47.4%, 18/38), nausea (44.7%, 17/38), proteinuria (36.8%, 14/38), vomiting (34.2%, 13/38), and hypoproteinemia (31.6%, 12/38).

**TABLE 3:** TRAEs in 38 patients.

	RC48 + PD‐1 inhibitor (*n* = 30)	RC48 alone (*n* = 8)
Anemia	70.0% (21/30)	75.0% (6/8)
Anorexia	60.0% (18/30)	50.0% (4/8)
Hypoesthesia	50.0% (15/30)	62.5% (5/8)
Myelosuppression	50.0% (15/30)	37.5% (3/8)
Nausea	50.0% (15/30)	25.0% (2/8)
Asthenia	46.7% (14/30)	75.0% (6/8)
Alopecia	43.3% (13/30)	62.5% (5/8)
Proteinuria	43.3% (13/30)	12.5% (1/8)
Vomiting	40.0% (12/30)	12.5% (1/8)
Hypoalbuminemia	36.7% (11/30)	12.5% (1/8)
Leukopenia	33.3% (10/30)	12.5% (1/8)
Acid regurgitation	33.3% (10/30)	12.5% (1/8)
Hyponatremia	26.7% (8/30)	37.5% (3/8)
γ‐Glutamyl transferase increased	26.7% (8/30)	12.5% (1/8)
Creatinine level increased	26.7% (8/30)	12.5% (1/8)
Apolipoprotein A1 level decreased	23.3% (7/30)	25.0% (2/8)
Urea nitrogen increased	20.0% (6/30)	12.5% (1/8)
Neutrophil count decreased	20.0% (6/30)	0
Positive for fecal occult blood test	16.7% (5/30)	0
Apolipoprotein B level increased	16.7% (5/30)	12.5% (1/8)
Triglyceride increased	16.7% (5/30)	50.0% (4/8)
Aspartate aminotransferase increased	13.3% (4/30)	0
Cholesterol level increased	13.3% (4/30)	50.0% (4/8)
Blood thyroid‐stimulating hormone increased	13.3% (4/30)	12.5% (1/8)
Venous blood glucose increased	13.3% (4/30)	12.5% (1/8)
Flatulence	13.3% (4/30)	12.5% (1/8)
Alanine transaminase increased	10.0% (3/30)	12.5% (1/8)
Lactate dehydrogenase increased	10.0% (3/30)	12.5% (1/8)
Myalgia	10.0% (3/30)	0
Astriction	10.0% (3/30)	0
Dysphagia	6.7% (2/30)	0
Bellyache	6.7% (2/30)	0
Reduced platelet count	6.7% (2/30)	0
Direct bilirubin level increased	3.3% (1/30)	12.5% (1/8)
Diarrhea	3.3% (1/30)	0
Blood thyroid‐stimulating hormone increased	3.3% (1/30)	0
Vascular neuropathic headache	3.3% (1/30)	0
Immune‐related AEs		
Rash	3.3% (1/30)	‐

*Note*: Data are presented as *n* (%). The table includes Grade 1 and 2 TRAEs that occurred in all the patients. No Grade ≥3 AEs were observed. And TRAEs in patients who received RC48‐ADC monotherapy are listed separately.

Abbreviation: TRAE, treatment‐related adverse event.

During the study's observation period, seven patients (18.4%) continued treatment, while 31 discontinued. The primary reasons for discontinuing RC48‐ADC were disease progression in 17 patients (45.9%), adverse events in five patients (13.2%), a change in regimen for financial reasons in six cases (13.2%), an exacerbation of underlying disease in two patients, and one patient (2.6%) discontinued treatment.

Specifically, among the 30 patients who received combination therapy, six patients continued treatment, 12 patients had disease progression, and five patients discontinued the treatment due to adverse events (one patient changed treatment regime due to intolerable hypoesthesia, one patient only stopped RC48‐ADC because of intolerable hypoesthesia, one patient only stopped RC48‐ADC due to intolerable gastrointestinal symptoms, one patient only stopped RC48‐ADC due to elevated creatinine levels up to 200 μmol/L, and one patient stopped all the antitumor therapy due to intolerable gastrointestinal symptoms), five patients changed treatment due to economic reasons, one patient stopped treatment due to aggravation of atrial fibrillation, and one patient voluntarily abandoned further treatment. Among the eight patients who received RC48‐ADC monotherapy, one patient continued treatment, five patients showed disease progression, one patient stopped treatment due to recurrent pancreatitis, and one patient changed treatment regime due to economic reasons. Based on the standard dose of 2.0 mg/kg, one patient had the initial RC48‐ADC dose reduced to 1.5 mg/kg and one patient had the initial dose reduced to 1.0 mg/kg after clinical evaluation. And one patient only discontinued the RC48‐ADC due to intolerable gastrointestinal symptoms in the seventh cycle and subsequently received tislelizumab monotherapy.

## 4 DISCUSSION

Recent years have seen remarkable achievements with the targeted HER2 RC48‐ADC in both gastric cancer and breast cancer.^22^, ^23^ Furthermore, this therapy has shown considerable potential in ongoing UC studies. The publication of a series of multicenter (randomized controlled trial) RCT research results has elevated RC48‐ADC beyond the limitations of traditional anti‐HER2 targeted therapy, thus offering a new alternative for patients with mUC. Nonetheless, due to the limited conditions of the clinical research participants, the study population often fails to adequately represent the patient population seen in actual clinical practice. As a response to this, we undertook a real‐world study to assess the effectiveness and safety of RC48‐ADC in real clinical settings, aiming to provide additional evidence for clinical decision‐making, particularly in certain patient populations not included in clinical trials.

ORRs divided by pathological baseline characteristics are shown in Figure S1. The confidence intervals observed in this figure may be attributable to the small cohort size. At a given confidence level of 95%, the width of the confidence intervals increases as the sample size decreases in each cohort after subgroups are delineated based on baseline pathologic characteristics. In this study, 38 patients with mUC were treated with RC48 alone or in conjunction with PD‐1 inhibitors. This population involved patients of advanced age, those having received multiple lines of therapy, and those with unfavorable baseline characteristics. The outcomes revealed ORR of 63.2%, a DCR of 89.5%, a median PFS of 8.2 months, a 6‐month PFS rate of 63.2%, a 12‐month PFS rate of 34.1%, and a median OS that was not reached, with a 12‐month OS rate of 76.7%. This study included seven cases (18.4%; Table 1) of ECOG PS2 patients, of which three patients achieved PR, two patients had SD, and two patients had PD. The median PFS was 9.6 months, higher than the overall level of 8.2 months, and the incidence of adverse reactions did not increase. This may be attributed to the heterogeneity of PS2 patients and the subjectivity of ECOG PS evaluation.^24^ Generally, poorer PS leads to lower chemotherapy response rates and shorter PFS.^25^ In this study, PS was determined by a combination of tumor disease progression and underlying diseases. In patients whose PS was determined by comorbidities rather than cancer progression, better prognosis may be demonstrated. In addition, a multicenter, retrospective study (GOIRC‐2018‐01) showed a longer time to progression in non‐small cell lung cancer patients whose PS resulted from the coexistence of comorbidities (median PFS 5.6 months), compared to the group without comorbidities and with worse performance resulting from cancer (median PFS 1.8 months).^26^ In the future, the efficacy and safety of ECOG PS two patients need to be further validated in prospective clinical trials.

In this study, eight patients who received RC48 monotherapy presented an ORR of 50.0%, numerically in line with the combined outcomes of the RC48‐C005 and RC48‐C009 studies (50.5%)^17^ and the RC48‐C011 study (26.3%).^16^ It exceeded the result presented by Chen et al. (38.8%).^27^ Moreover, the median PFS (6.9 months) was numerically superior to the outcomes of the combined analysis of the RC48‐C005 and RC48‐C009 studies (5.9 months), the outcomes of the RC48‐C011 study (5.5 months),^16^ and the data of Chen et al. (5.4 months).^27^ The study also included 30 patients who received RC48 in combination with a PD‐1 inhibitor, showing an ORR of 66.7%, lower than the RC48‐C014 study result (76.7%),^19^ but higher than that reported by Chen et al. (38.8%).^27^ The median PFS of 8.2 months was lower than the RC48‐C014 study result (9.2 months) but similar to the real‐world data from Chen et al. (8.5 months).^27^ Interestingly, the impressive ORR and PFS exhibited by RC48 monotherapy were surprising findings. However, the ORR and PFS data from the RC48 monotherapy regimen and combined treatment did not show statistically significant differences, consistent with the findings of Chen et al.,^27^ although further prospective studies are necessary. Additionally, the ORR of RC48‐ADC as a first‐line treatment was 71.4%, higher than its ORR as a second‐line and subsequent treatment at 58.3%. However, the PFS of RC48‐ADC as a first‐line treatment was 6.3 months, shorter than its PFS as a second‐line and subsequent treatment at 8.2 months. The primary reason for this discrepancy with the conclusions of Chen et al.^27^ stems from the fact that 4 of the 14 patients who received RC48‐ADC as a first‐line treatment switched to alternative treatments for financial reasons without substantial disease progression being observed, thereby affecting the PFS results. A firm conclusion cannot be made given limitations of this real‐world study, which includes small sample size, lack of control design, various line of therapy setting, missing HER2 IHC level in half of the subjects, etc. Chen et al.'s study included 18 patients with mUC treated with a combination of immunotherapy and RC48, including a total of five different combination therapies of PD‐1 inhibitors and PD‐L1 inhibitors. They discussed the overall effectiveness and safety of immunotherapy combined with RC48 in the treatment of mUC from a broad perspective. In comparison with Chen et al., we analyzed one aspect of immunotherapy, specifically PD‐1 inhibitors, and included 30 patients to discuss the effectiveness and safety of PD‐1 inhibitors combined with RC48 in the treatment of mUC. Including a larger sample size and a more homogenous treatment regimen, the credibility increased to some extent. In addition, we discussed the treatment effects of two different PD‐1 inhibitors combined with RC48, while there has been no specific analysis of the combination of RC48 with different ICIs. Although our final data differences were not statistically significant, it also suggests a new research direction, which is the optimal combination therapy using RC48 for mUC patients. In the future, whether the combination of RC48 with PD‐1 inhibitors or the combination of RC48 with PD‐L1 inhibitors will benefit patients more may become a new research direction.

For a considerable duration, numerous studies have affirmed that HER2 expression is not only indicative of enhanced disease invasiveness and poorer prognosis but also presents as a potential therapeutic target.^28^, ^29^, ^30^ There are clinical trials suggesting that the efficacy of anti‐HER2 ADC seems to be related to the level of HER2 expression. The efficacy of anti‐HER2 ADC T‐DXd was evaluated in the DAISY phase 2 clinical trial for patients with metastatic breast cancer with HER2 overexpression (72 cases, Cohort 1), HER2 low expression (74 cases, Cohort 2), and HER2 non‐expression (40 cases, Cohort 3). The results showed that the mPFS was longer in Cohort 1 at 11.1 months, Cohort 2 had an median PFS of 6.7 months, and Cohort 3 had an median PFS of 4.2 months.^31^ The interim results of the DESTINY‐PanTumor02 (DP‐02) trial included 41 patients with urothelial cancer, with an overall response rate (ORR) of 56.3% in 16 patients with HER2 3+ compared to an ORR of 35.0% in 20 patients with HER2 2+.^32^ Additionally, Klümper et al.'s study showed that the clinical benefit of EV strongly depends on membranous NECTIN‐4 expression, and the lack or low expression of NECTIN‐4 is significantly associated with shortened PFS of EV.^33^ Traditional anti‐HER2 therapies such as trastuzumab and lapatinib have demonstrated limited clinical benefit in treating UC.^34^, ^35^ In China, RC48‐ADC has received approval for treating mUC patients with HER2 overexpression (HER2 IHC2+ and FISH+ or IHC3+) who exhibit progression post standard chemotherapy. According to Zhou et al.,^36^ the rate of HER2 overexpression (IHC2+ and 3+) among the Chinese UC population stands at 44%, with HER2‐positive patients constituting 51% of bladder cancer cases and 38% of upper tract UC (UTUC) cases. Even without considering HER2 status, survival in the entire population has been improved by RC48‐ADC. With the advent of RC48‐ADC as a new generation of targeted therapy, HER2 expression no longer signifies a poor prognostic factor for UC. Instead, it is associated with better prognosis in patients treated with RC48‐ADC, but this has not been confirmed in controlled prospective studies. Consequently, it has been proposed that UC patients be classified as HER2‐expressive (IHC 1+, 2+, 3+) and negative (IHC 0), with the role of fluorescence in situ hybridization (FISH) deemed limited. Following this perspective, the ORR was found to be 70.6% (12/17) among 17 HER2‐expressive patients, while the best response evaluation in one HER2‐negative patient was SD. Specifically, five HER2‐expressive patients treated with RC48 monotherapy had PR as the best response evaluation, while one HER2‐negative patient treated with RC48 monotherapy had SD as the best response evaluation. Moreover, 9 out of 12 HER2‐expressive patients received the combination of RC48 and the PD‐1 inhibitor achieved PR. Based on the actual situation, there may be the following reasons for HER2 deficiency: First, many patients have undergone radical resection at their initial visit, making it difficult to obtain specimens for HER2 testing again. Second, if samples are taken for too long in the past, there may be attenuation in the samples, and considering accuracy issues, retesting is abandoned. Furthermore, the patient underwent HER2 testing in hospitals that did not participate in this study, but the HER2 results between different hospital medical systems were not synchronized, resulting in unclear results. In addition, HER2 testing is a self‐funded project, and although the cost is not too high, there are still a few patients who choose not to undergo HER2 testing after a clear diagnosis of UC due to economic reasons. We actively support patients to undergo HER2 testing as much as possible to clarify HER2 levels when conditions permit.

Another key objective of real‐world studies involves investigating the treatment of specific patient populations that are typically underrepresented in clinical trials. In our study, 30 patients received a regimen combining RC48 and PD‐1 inhibitors. These PD‐1 inhibitors included tislelizumab in 18 cases and toripalimab in 12 cases. And there was no difference in ORR between PD‐1 inhibitor‐naive patients and PD‐1 inhibitor‐exposed patients (*p* = 0.44). The ORR for patients who were administered RC48‐ADC in combination with toripalimab was 75.0%, along with a median PFS of 8.9 months. Among them, the previous treatment with immunotherapy resulted in an ORR of 66.7% (4/6), while the ORR for those who did not receive immunotherapy was 83.3% (5/6), with a *p*‐value of 1.00. Meanwhile, for patients treated with RC48‐ADC in combination with tislelizumab, the ORR was 61.1% with a median PFS of 6.8 months. Among them, the ORR for those who had previous immunotherapy was 50.0% (4/8), while the ORR for those who had not received immunotherapy was 70.0% (7/10), with a *p*‐value of 0.63. Numerically, though the ORR and PFS data of the RC48‐ADC plus toripalimab regimen were superior to those of the RC48‐ADC plus tislelizumab regimen, the difference was not statistically significant. This was consistent with the conclusions reached in a Phase II randomized clinical study that head‐to‐head compared sintilimab and pembrolizumab as the first‐line treatment in advanced NSCLC (CTONG1901).^37^ Furthermore, the results also showed that whether patients had received immunotherapy before or not had no statistically significant impact on the ORR of the combination treatment mentioned above. Although there were some differences in the numbers, the small sample size and individual patient variations could lead to significant fluctuations in the efficacy rates, making it impossible to determine superiority or inferiority based solely on the numbers. Therefore, it can still be concluded that the effectiveness of the two groups is similar. Therefore, there is a need for large sample prospective studies to further investigate the optimal treatment regime for different patient populations utilizing RC48‐ADC. At present, the PD‐1 inhibitors approved in China for treating UC primarily include tislelizumab and toripalimab. Undoubtedly, the comprehensive decision‐making regarding the use of PD‐1 inhibitors in a clinical setting requires physicians to make choices based on a multitude of factors.

In this study, no patients experienced Grade 3 or higher TRAEs with either RC48‐ADC alone or RC48‐ADC in combination with the PD‐1 inhibitors tislelizumab or toripalimab. The most frequently observed TRAEs included anemia (71.1%, 27/38), nausea (57.9%, 22/38), asthenia (52.6%, 20/38), hyperalgesia (52.6%, 20/38), myelosuppression (47.4%, 18/38), alopecia (47.4%, 18/38), additional instances of nausea (44.7%, 17/38), proteinuria (36.8%, 14/38), vomiting (34.2%, 13/38), and hypoproteinemia (31.6%, 12/38). Anemia was the most prevalent adverse reaction observed in this study, differing from the findings of the RC48 series studies. This discrepancy may be attributable to the multifactorial etiology of anemia in patients with malignancies.^38^ Patients with genitourinary tumors may experience disease‐related bleeding, tumor infiltration, and myelosuppression. Myelosuppression and nephrotoxicity induced by antitumor therapy can suppress immunity and inhibit erythropoiesis. Moreover, poor appetite and nutritional deficiencies can also amplify the development of anemia. Consequently, the actual incidence of RC48‐ADC treatment‐related anemia might be lower than observed in this study. Furthermore, patients with poor ECOG PS, anemia, distress, pain, and those undergoing oncological treatment were found to be at significantly higher risk for cancer‐related fatigue.^39^ In alignment with the findings of previous studies, the incidence of TRAEs was higher in patients who received combination therapy compared to those who received RC48 monotherapy. The correlation between the incidence of TRAEs and the type of PD‐1 inhibitors used in combination therapy merits further exploration.

Notable limitations of this study were its retrospective nature, a limited sample size, incomplete HER2 expression and PD‐1 data, and heterogeneous prior treatment regimens among patients. Due to geographical restrictions, a significant number of patients initially received treatment in hospitals other than the three selected for this study. Furthermore, limitations of medical record systems allowed us to only access electronic medical records indicating that “in combination with HER2 expression results and relevant clinical trial data, RC48‐ADC was recommended, and after detailed communication with the patients and their families, they agreed and signed the informed consent forms.” Hence, the HER2 expression status of 20 patients (52.6%) remained uncertain, which restricted our ability to conduct a more comprehensive stratified analysis based on HER2 expression status.

## 5 CONCLUSION

This study provides a summary of the real‐world experiences of patients with mUC treated with RC48 either alone or in combination with PD‐1 inhibitors, including a subset of elderly patients and those with an ECOG score of ≥2. The results demonstrated that RC48 monotherapy or RC48 combined with PD‐1 inhibitors exhibits promising efficacy and manageable safety profiles for mUC patients in China. The effectiveness and safety were in line with the outcomes of several pivotal clinical trials, suggesting that RC48‐ADC could be further explored for application among the Chinese population. In our study, a substantial number of patients discontinued further treatment due to financial constraints. However, since RC48‐ADC was included in the national medical insurance system's drug catalog in January 2023, a larger number of mUC patients will be able to afford this treatment. Future prospective studies with larger sample sizes could be conducted to better evaluate the efficacy and safety of different RC48 combination regimens in mUC patients in China. A Phase III clinical trial of RC48‐ADC in combination with toripalimab as a first‐line treatment for mUC is currently underway (NCT05302284).

## AUTHOR CONTRIBUTIONS

**Jingwei Xu:** Conceptualization (equal); formal analysis (equal); writing – original draft (equal). **Hongqiao Zhang:** Conceptualization (equal); data curation (equal); writing – original draft (equal). **Li Zhang:** Writing – review and editing (supporting). **Xiufeng Chu:** Writing – review and editing (supporting). **Yu Li:** Writing – review and editing (supporting). **Guangyuan Li:** Data curation (supporting); formal analysis (supporting). **Caiyun Nie:** Data curation (supporting); formal analysis (supporting). **Meng Wang:** Data curation (supporting); formal analysis (supporting). **Yanwei Guo:** Conceptualization (lead); writing – review and editing (lead).

## FUNDING INFORMATION

The authors declare that no funds, grants, or other support were received in the preparation of this manuscript.

## CONFLICT OF INTEREST STATEMENT

The authors indicate no financial or non‐financial interests.

## ETHICS APPROVAL STATEMENT

This study was approved by the Ethics Committee of the Fifth Affiliated Hospital of Zhengzhou University, and the study's procedures complied with the ethical standards outlined in the World Medical Association Declaration of Helsinki.

## PATIENT CONSENT STATEMENT

The requirement for individual informed consent was waived by the committee due to the retrospective nature of the study.

## PERMISSION TO REPRODUCE MATERIAL FROM OTHER SOURCES

NA.

## CLINICAL TRIAL REGISTRATION

NA.

## Supporting information


Figure S1.
Figure S2.

## Data Availability

The datasets generated during the current study are available from the corresponding author upon reasonable request.
