# Supplementary material for: Real‐world effectiveness and safety of RC48‐ADC alone or in combination with PD‐1 inhibitors for patients with locally advanced or metastatic urothelial carcinoma: A multicenter, retrospective clinical study
Source: Cancer Med. 2023 Nov 7;12(23):21159–71. doi: 10.1002/cam4.6680 (PMC10726858; doi:10.1002/cam4.6680)
Supplement: Supplementary file 1 — Figure S1. Figure S2. [file CAM4-12-21159-s001.docx]

**Supplementary Figure S1**


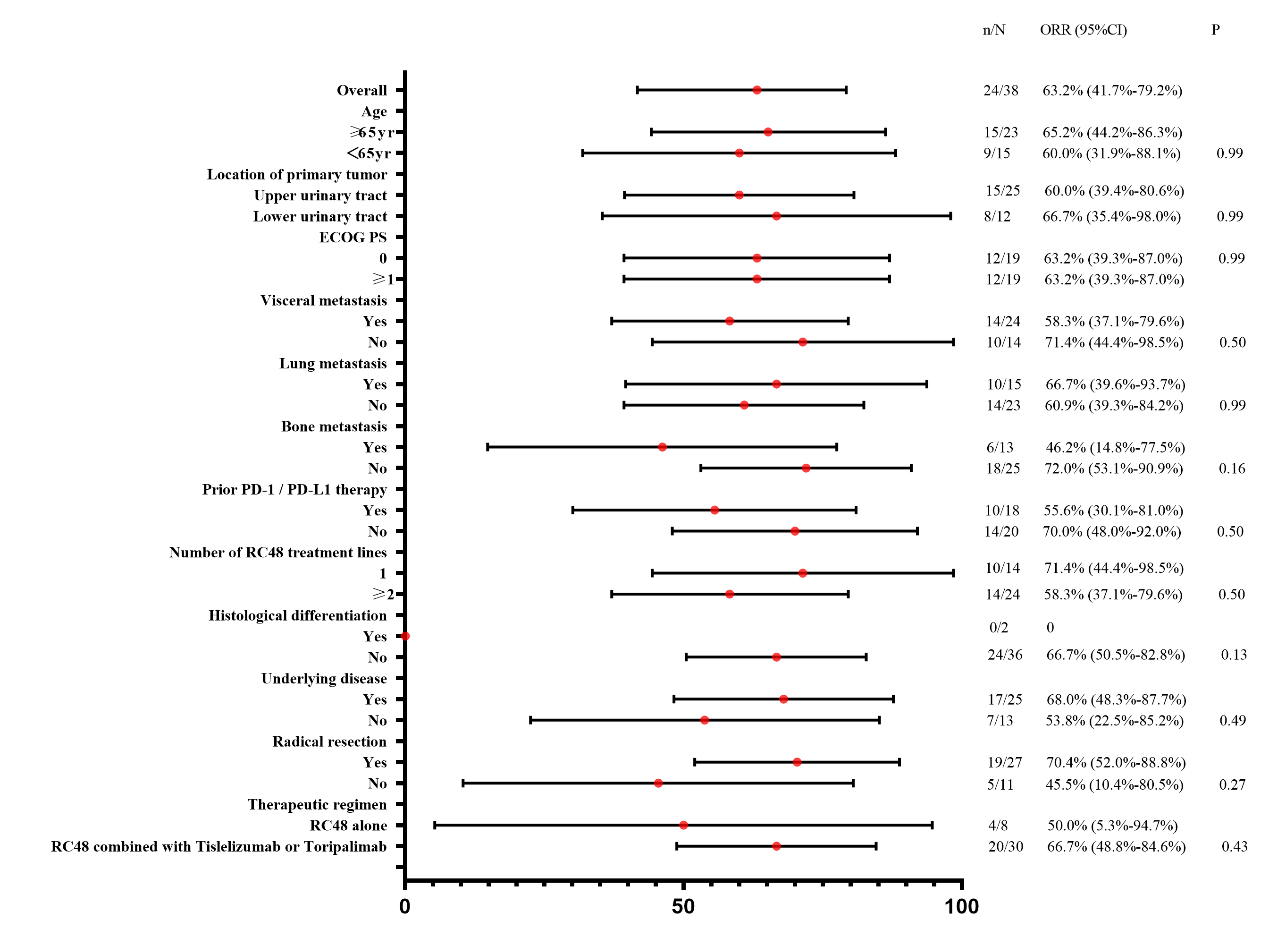


**Supplementary Figure S2**


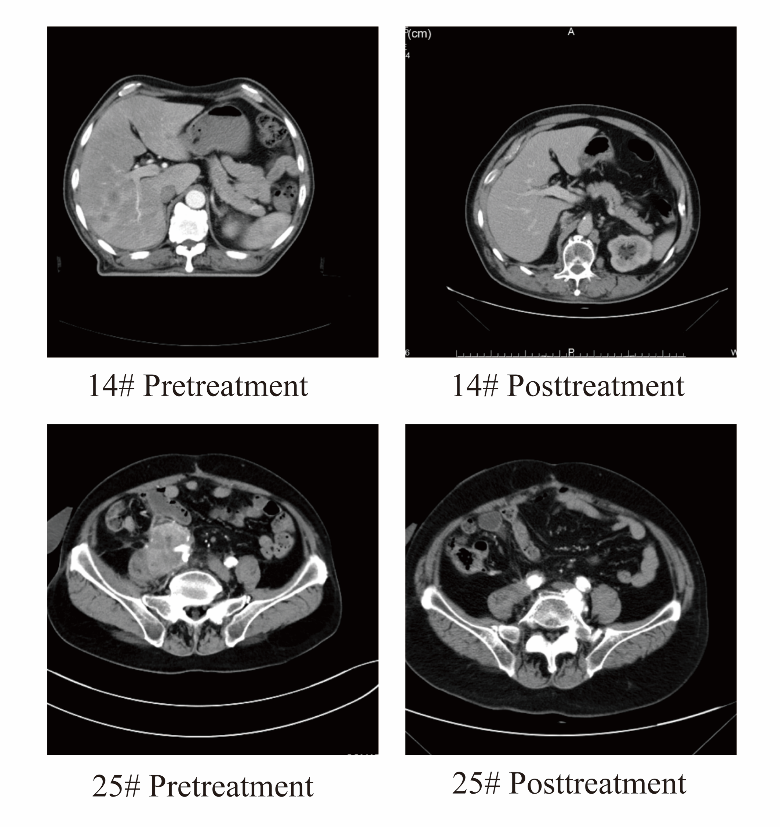


**Figure legends**

**Figure S1.** Forest plot of subgroup ORR delineated based on baseline disease characteristics. We excluded a patient with a primary lesion in both the upper and lower urinary tracts to compare the ORR of the two subgroups.

**Figure S2.** The representative pretreatment and posttreatment imaging with responses in metastases of 2 different patients.
